# A bidirectional two-sample Mendelian randomization study to evaluate the relationship between psoriasis and interstitial lung diseases

**DOI:** 10.1186/s12890-024-03146-y

**Published:** 2024-07-09

**Authors:** Lixia Yue, Yihe Yan, Shushan Zhao

**Affiliations:** 1 Department of Rheumatology, Shaoxing People’s Hospital, Shaoxing, Zhejiang 312000 China; 2 Department of Critical Care Medicine, Shaoxing People’s Hospital, Shaoxing, Zhejiang 312000 China

**Keywords:** Psoriasis, Psoriatic arthritis, Interstitial lung disease, Mendelian randomization, Causal relationship

## Abstract

**Background:**

Prior observational studies have suggested a potential direct link between psoriasis (PSO) and interstitial lung disease (ILD). Consequently, we applied Mendelian randomization (MR) to further evaluate the bidirectional causal relationships between PSO and its different phenotypes [psoriatic arthritis (PSA)/psoriasis vulgaris (PSV)] and ILD.

**Methods:**

Data regarding PSO/PSA/PSV and ILD were sourced from publicly accessible genome-wide association studies (GWAS) databases, focusing on European populations. We used five algorithms— MR Egger, weighted median, inverse-variance weighted (IVW), simple mode, and weighted mode— to evaluate the causal relationships between PSO/PSA/PSV and ILD, with a primary emphasis on the IVW method.

**Results:**

The analysis indicated a potential association between PSA and an elevated risk of ILD [IVW odds ratio (OR): 1.035 (95% CI 1.008, 1.064; *P* = 0.012)], with no evidence of a direct relationship between total PSO and PSV with ILD. Conversely, no substantial evidence emerged from the reverse MR analysis to suggest that ILD significantly affects total PSO or the specific PSA/PSV phenotypes.

**Conclusion:**

Our findings provide genetic evidence supporting the notion that PSA may be a contributory risk factor for ILD. Further investigations are warranted to explore the underlying mechanisms of this potential causal relationship between PSA and ILD.

**Supplementary Information:**

The online version contains supplementary material available at 10.1186/s12890-024-03146-y.

## Introduction

Psoriasis (PSO) is a chronic skin condition marked by rapid skin cell turnover, leading to red, scaly patches covered with silver-white scales [1]. It includes phenotypes such as psoriasis vulgaris (PSV), psoriatic arthritis (PSA), guttate psoriasis (GP), and pustular psoriasis (PP), with PSV being most common and PSA affecting skin and joints [2, 3]. Globally, PSO affects approximately 100 million individuals, with 1.3–34.7% potentially developing PSA, which can result in joint deformities [4]. Although PSO is usually not life-threatening, it can significantly impact the psychological health of patients. Moreover, PSO is associated with various comorbidities, including neurological, cardiovascular diseases, and psychiatric complications [5].

Interstitial lung disease (ILD) encompasses a group of lung diseases primarily impacting the pulmonary interstitium, characterized by lung interstitium inflammation and fibrosis [6]. It is estimated that over 200 diseases can cause ILD, including rare diseases (such as lymphangioleiomyomatosis), lung diseases (such as idiopathic pulmonary fibrosis), and multisystem diseases (such as systemic sclerosis) [7]. The incidence of ILD varies by age, gender, and ethnicity, and can progress from reversible to fatal stages without early intervention. Despite optimal treatment, ILD can be fatal. Therefore, early diagnosis and treatment of ILD are crucial, and eliminating causative factors can significantly alleviate or even cure the disease [7, 8].

Previous studies suggest that the prevalence of pulmonary fibrosis among patients with PSO is comparable to that in the general population. The occurrence of ILD in PSO patients is often attributed to drug-induced pneumonia, a consequence of the immunosuppressive medications used in PSO treatment [9]. However, Ishikawa and colleagues found ILD and PSO often co-occur in untreated patients at Mount Sinai Hospital, analyzing data from 21 ILD-PSO patients and noting 63.6% hadn’t received immunosuppressives [10]. Similarly, Bargagli et al. reviewed six patients with PSA and ILD, discovering half were diagnosed with PSA before ILD, the rest simultaneously developing symptoms [11]. These findings collectively hint at a possible causal link between PSO/PSA and ILD.

Mendelian randomization (MR) is a novel method that uses genetic variations as instrumental variables (IVs), leveraging the principle of genetic segregation to assess the causal relationship between exposure and outcomes [12]. This method utilizes the randomness of genetic variations to effectively control confounding factors and reverse causation interference. In this context, our study employed four pooled data from extensive genome-wide association studies (GWAS) public databases to conduct a bidirectional, two-sample MR analysis. The objective was to elucidate the bidirectional causal relationship between PSO and ILD, assessing the strength and direction of this association.

## Methods

### Data sources and study design

**Fig. 1:**
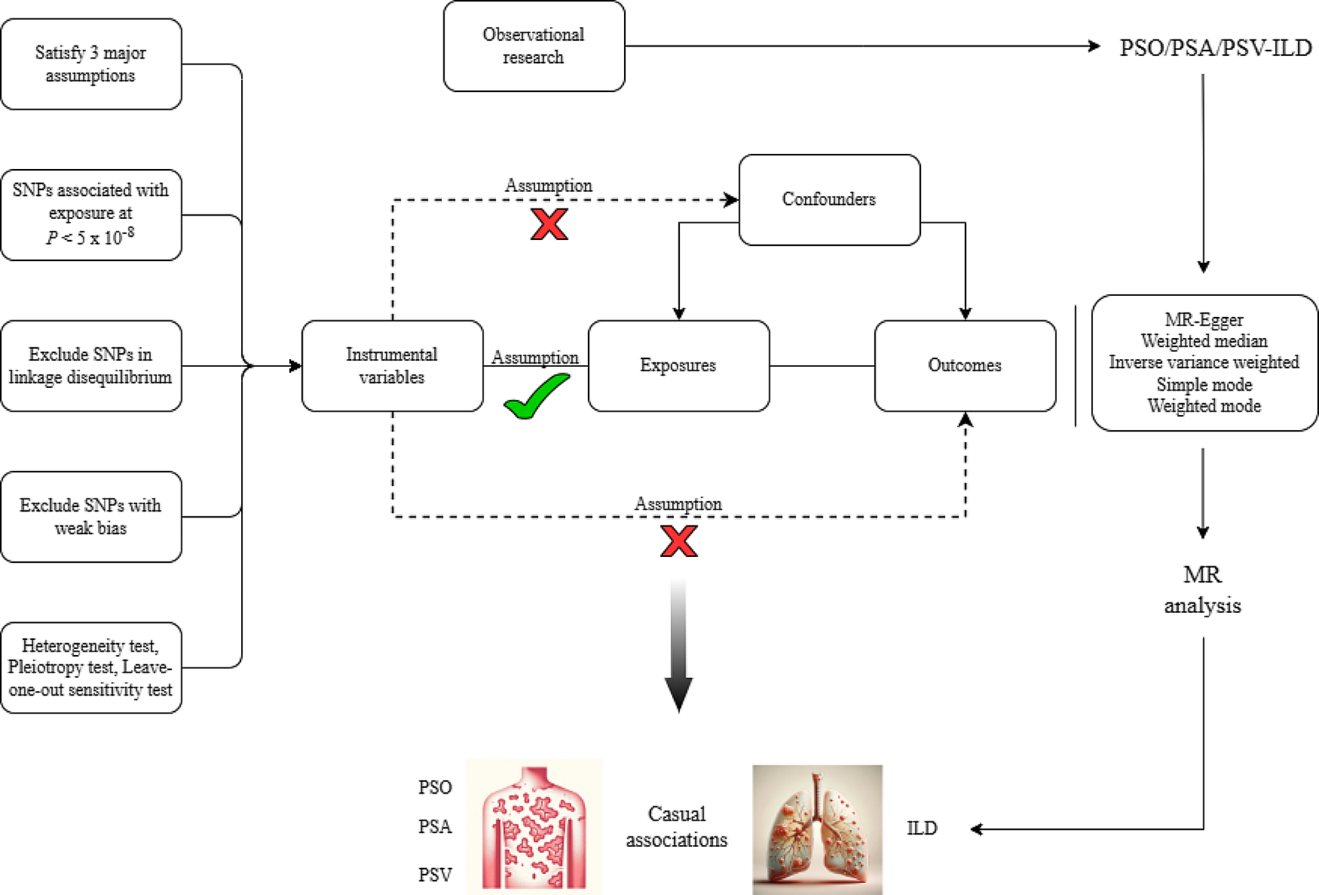
The MR analysis of causal associations between PSO (including PSA/PSV) and ILD. The green checks suggest a correlation between IVs and exposure, while the red crosses indicate no correlation with confounders or outcomes. *PSO* psoriasis, *PSA* psoriatic arthritis, *PSV* psoriasis vulgaris, *ILD* interstitial lung disease

We utilized publicly accessible GWAS ( https://gwas.mrcieu.ac.uk/) [13]: PSO data (5,314 cases, 457,619 controls; SNPs = 9,851,867) from the UK Biobank, PSA data (1,637 cases, 212,242 controls; SNPs = 16,380,462) and PSV data (2,802 cases, 212,242 controls; SNPs = 16,380,459) from the FinnGen Biobank [14], and ILD data (2,267 cases, 467,560 controls; SNPs = 24,192,245) from the European Bioinformatics Institute (EBI) [15]. To minimize bias from race-related confounders, we limited the genetic background of the study population to European ancestry (Supplementary Table 1). Figure 1 illustrates the data extraction workflow for this study in detail. Since all data were publicly available, additional ethical approval was not required. The diagnoses of PSO, PSA, PSV, and ILD were meticulously based on the International Classification of Diseases, 10th Revision (ICD-10) criteria.

### Instrumental variables selection and quality control

Multiple single nucleotide polymorphisms (SNPs) representing genetic variations were chosen as IVs for two-sample MR analysis. We adhered to three critical assumptions: (1) IVs are directly associated with the exposure; (2) IVs are independent of any confounders; (3) IVs influence the outcome solely through the exposure [16]. To effectively avoid potential linkage disequilibrium interference among SNPs, the following criteria were used to identify SNPs: (1) significant threshold related to IV: *P* < 5 × 10^-8; (2) r² < 0.1; (3) physical distance between genes = 10,000 kb. For PSO, which had numerous IVs, r² < 0.05 was set. To ensure sufficient correlation between IVs and exposure, we incorporated the *F* statistic to prevent any bias from weak IVs [17]. The *F* statistic for each SNP was calculated using the following equation: *F* = R^2^ (N-2)/(1-R^2^). R^2^ represents the variance of each collected IV on MSCTD. To calculate R² for each IV, we used the following formula: R^2^ = 2β^2^EAF(1-EAF)/[2β^2^EAF(1-EAF) + (se(β))^2^NEAF(1-EAF)], where EAF is the effect allele frequency, β is the estimated genetic effect on exposure, N is the sample size of the GWAS, and se is the standard error of the genetic effect. IVs with an F statistic less than 10 were considered weak instruments and were excluded from the MR analysis. Finally, 70 SNPs, 57 SNPs, and 78 SNPs were made available for any PSO, PSA, and PSV, respectively. For reverse MR analysis, 10 SNPs were made available for ILD (Supplementary Tables 2–7).

### Statistical analysis

The inverse variance weighted (IVW) method operates under the assumption that all genetic variants are valid instrumental variables, positioning it theoretically closest to an accurate estimate of the true effect. However, should this assumption be contravened, the resulting effect estimate may be subject to bias. MR Egger allows for the detection of genetic confounding but at the cost of lower efficiency. The weighted median provides robustness against invalid instrumental variables, yet it may not be as precise as IVW. The simple mode, lacking weighting, could lead to inefficiency. The weighted mode exhibits high robustness against extreme values, yet identifying a clear mode can be challenging when multiple common effect sizes are present. Therefore, the IVW algorithm was used as the primary method, combined with four other methods including MR Egger, weighted median, simple mode, and weighted mode to estimate bidirectional causal relationships between PSO, PSA, PSV, and ILD, assessing the effect size using odds ratio (OR) and 95% confidence interval (CI). Additionally, the MR-PRESSO method was used to detect and correct pleiotropy caused by instrumental variable heterogeneity or outliers. This method first calculated the global and specific residual sum of squares for each SNP and then used these values to identify potential outlier instrumental variables. After identifying outliers, MR-PRESSO corrected the causal estimate by excluding these outliers [18]. For heterogeneity testing, Cochrane’s Q-statistic was used, with *P* < 0.05 considered significant, indicating significant heterogeneity. In such cases, we opted for a random-effect model for estimating the effect size and subsequent analysis [19]. MR Egger regression was used to assess and adjust for potential horizontal pleiotropy. The presence of horizontal pleiotropy was determined based on the intercept of the MR Egger regression results and its significance level. Despite these efforts, the specter of residual confounding by pleiotropy remained, necessitating a leave-one-out analysis to determine the influence of individual SNPs on the overall association. All statistical analyses were performed using the statistical software R (version 4.3.0), along with the TwoSampleMR (version 0.5.7) and MR-PRESSO (version 1.0) packages.

## Results

### Effect of PSO/PSA/PSV on ILD

In our MR analysis examining the relationship between total PSO and ILD, significant heterogeneity was indicated (Cochrane’s Q-statistic p-value = 0.0091), necessitating the adoption of a random-effects model. Conversely, for PSA and PSV, no notable heterogeneity was observed, allowing for the application of a fixed-effect model. The MR-Egger pleiotropy test did not reveal any horizontal pleiotropy (*P* > 0.05). Nevertheless, the PRESSO global test identified minor IVs bias, leading to the exclusion of three anomalous SNPs (rs9266075, rs1003879, rs28732090) as outlined in Table 1.

**Fig. 2:**
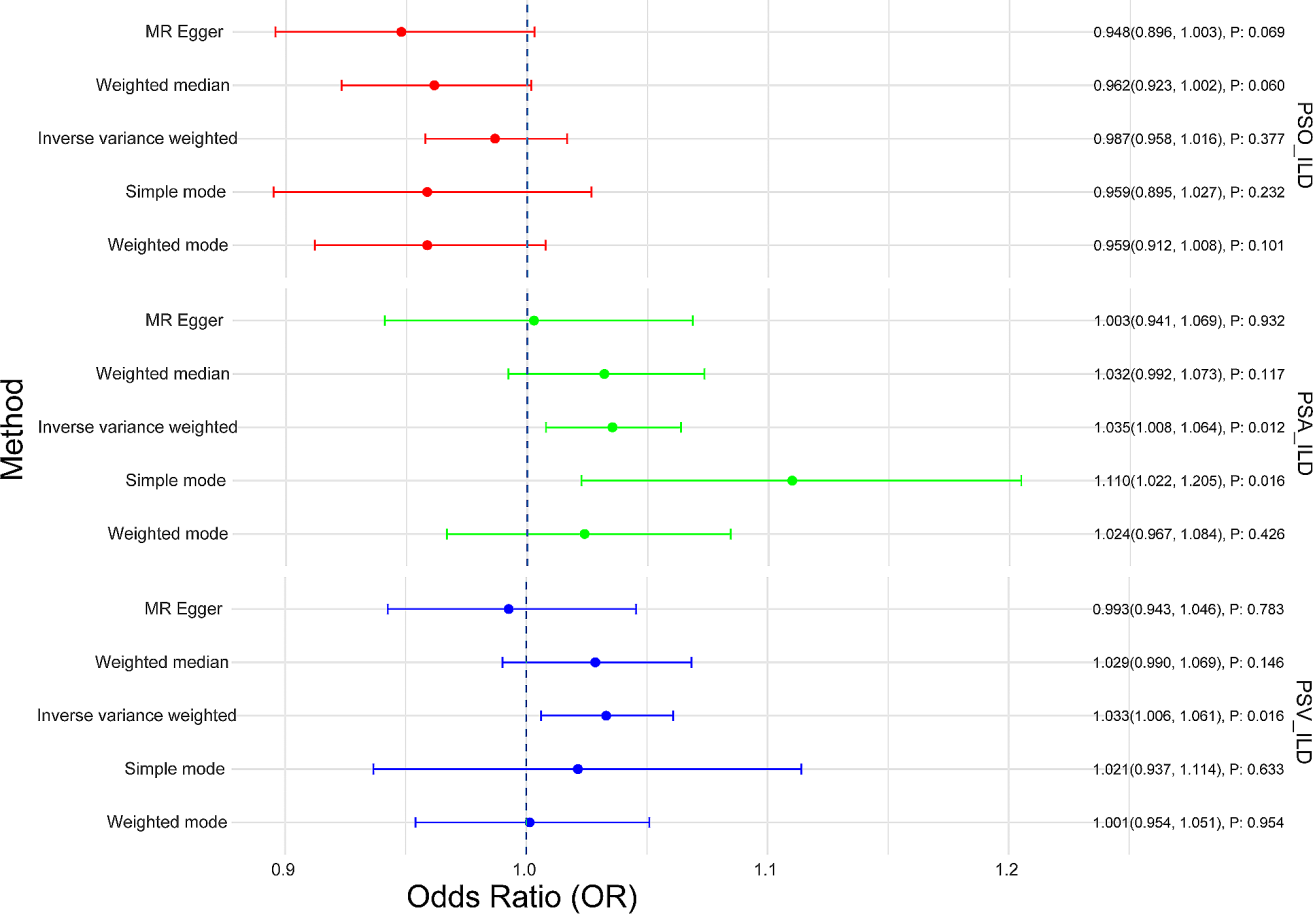
The risk association between PSO/PSA/PSV and ILD in a forest plot. *ILD* interstitial lung disease, *PSO* psoriasis, *PSA* psoriatic arthritis, *PSV* psoriasis vulgaris

These IVs are consequently considered robust for evaluating the causal links between PSO/PSA/PSV and ILD. To evaluate these relationships, five algorithms were employed: MR Egger, weighted median, IVW (mainly method), simple mode, and weighted mode). The corresponding ORs for PSA and ILD were as follows: 1.003 (95% CI 0.941, 1.069; *P* = 0.932), 1.032 (95% CI 0.992, 1.073; *P* = 0.117), 1.035 (95% CI 1.008, 1.064; *P* = 0.012), 1.110 (95% CI 1.022, 1.205; *P* = 0.016), and 1.024 (95% CI 0.967, 1.084; *P* = 0.426), respectively. No causal relationship was found between PSO and ILD [IVW OR: 0.987 (95% CI 0.958, 1.016; *P* = 0.377)]. For the MR analysis between PSV and ILD, although the IVW method indicated a significant causal relationship [1.033 (95% CI 1.006, 1.061; *P* = 0.016)], however, the direction of the OR in the MR Egger method was inconsistent [0.993 (95% CI 0.943, 1.046; *P* = 0.783)], hinting at potential unmeasured confounders and therefore diminishing the robustness of these results (Fig. 2). Scatter plots (Supplementary Fig. 1) revealed a significant positive causal effect of PSA on ILD. Additionally, leave-one-out sensitivity analysis indicated that the association between PSA and ILD was not driven by any single SNP, suggesting the stability of the effect estimation (Supplementary Fig. 2). Collectively, these findings propose that PSA may elevate the risk of developing ILD.

**Table 1:** Sensitivity analysis

Exposure	Outcome	Number of IVs	Heterogeneity test		MR-Egger pleiotropy test		MR-PRESSO global outlier test			
			Q	*P*-value	Intercept	*P*-value	RSSobs	*P*-value	Outlier	
PSO	ILD	67	93.8155	0.0170	0.0091	0.2649	104.4780	0.0065	rs9266075, rs1003879, rs28732090	
PSA		57	58.8356	0.2105	0.0136	0.2829	68.8839	0.1640	None	
PSV		78	96.5476	0.1597	0.0169	0.0850	111.6612	0.0820	None	
ILD	PSO	8	2.4693	0.9294	0.0336	0.0418	19.5821	0.0300	rs35705950	
	PSA	10	1.8844	0.9844	0.0633	0.0235	20.1434	0.2819	None	
	PSV	10	4.1691	0.8416	0.0223	0.2426	9.7673	0.6294	None	

*Notes ILD* interstitial lung disease, *PSO* psoriasis, *PSA* psoriatic arthritis, *PSV* psoriasis vulgaris

### Effect of ILD on PSO/PSA/PSV

**Fig. 3:**
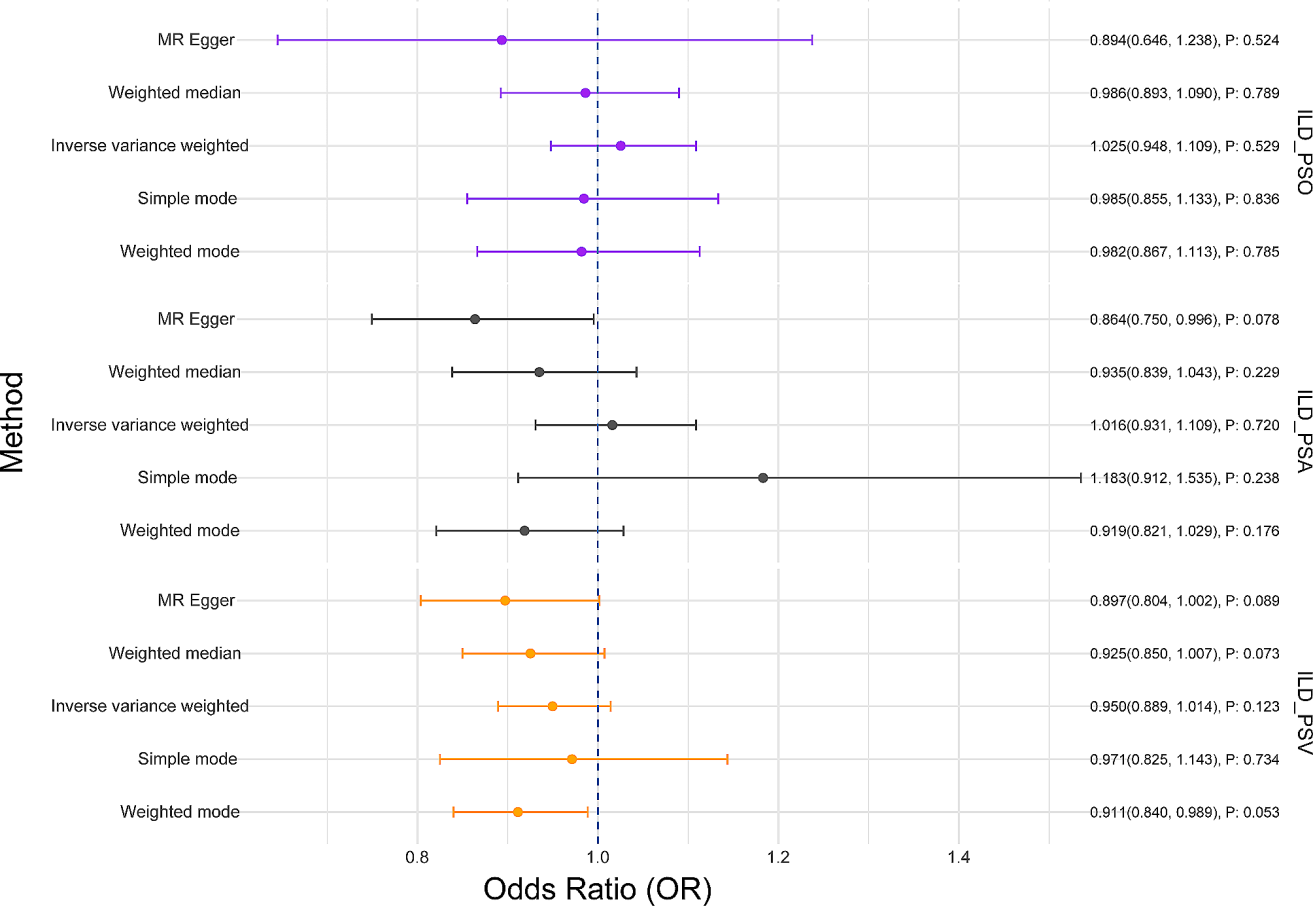
The risk association between ILD and PSO/PSA/PSV in a forest plot. *ILD* interstitial lung disease, *PSO* psoriasis, *PSA* psoriatic arthritis, *PSV* psoriasis vulgaris

Reverse MR analysis was conducted to investigate the potential causal relationship of ILD on PSO/PSA/PSV. The Cochrane’s Q statistic was utilized to assess heterogeneity, with results indicating no significant heterogeneity (*P* > 0.05). Consequently, a fixed-effect model was selected for the subsequent MR analysis (Table 1). Genetically predisposed higher ILD was not associated with a higher risk of PSO at any phenotype [IVW OR:1.025 (95% CI 0.948, 1.109; *P* = 0.529)], PSA [IVW OR: 1.016 (95% CI 0.931, 1.109; *P* = 0.720)], or PSV [IVW OR: 0.950 (95% CI 0.889, 1.014; *P* = 0.123)] (Fig. 3). Scatter plots and leave-one-out sensitivity analysis results are presented in Supplementary Figs. 3 and 4.

## Discussion

To our knowledge, this study represents the inaugural MR analysis exploring the bidirectional causal relationships between PSO and ILD. Our investigation centered on the bidirectional causal links between total PSO, PSA, and PSV in relation to the risk of developing ILD. The results indicated a notable association between PSA and an increased risk of ILD [IVW OR: 1.035 (95% CI 1.008, 1.064; *P* = 0.012)].

Previous literature on ILD occurrence in PSO patients is scarce, and when present, it is often overshadowed by the attribution of pulmonary complications to the toxicity of therapeutic drugs. Methotrexate, a widely used immunosuppressant for treating severe PSO and PSA, is frequently implicated, though the specifics of its pulmonary toxicity remain elusive [20–22]. Yasmeen et al. analyzed the medical records of 44 patients with diffuse parenchymal lung disease (DPLD) and PSO/PSA, finding that 27 had PSA (61%), and nearly a third had not previously received immunosuppressive treatment [23]. Additionally, various studies indicate an elevated prevalence of PSO alongside chronic obstructive pulmonary disease [24] and asthma [25], implying a direct link between PSO/PSA and pulmonary diseases beyond drug-induced effects. The causal relationship between PSO and ILD has only recently gained attention. Although PSO is generally considered benign in terms of mortality, the potential association with life-threatening diseases like ILD should not be underestimated. The majority of literature establishing their correlation comes from observational studies [10, 11, 26], which are difficult to avoid being influenced by confounding factors, selection bias, and information bias, thereby weakening the judgment of disease correlation. Moreover, even in cases of strong correlation, establishing the causal sequence is challenging. The exploration of causality between these two comorbidities is complex, given their shared risk factors like obesity [27, 28], smoking [29, 30], and autoimmune system irregularities [31, 32]. Therefore, MR analysis offers a more precise estimation of causal relationships. In this study, we identified a phenotype-specific effect of PSA on ILD, revealing that PSA, but not PSO or PSV, is causally associated with ILD outcomes. This finding delineates the differences between PSA and PSV, suggesting that beyond affected areas, they may also exhibit significant divergences in their pathological mechanisms.

Connective tissue diseases (CTD) comprise a range of disorders affecting the body’s connective tissue, often marked by immune system irregularities. ILD represents the most common and severe pulmonary complication within CTD, characterized by lung connective tissue being erroneously targeted by a dysregulated immune system, leading to chronic inflammation. This inflammation is further intensified by the infiltration of immune cells and the release of inflammatory mediators in the lungs [33, 34]. Given the autoimmune abnormalities and inflammatory responses in PSO/PSA, their association with ILD appears plausible. CD4 + Th17 cells and their cytokines, crucial in both PSO/PSA and ILD, may serve as a potential link between these conditions. We hypothesize that in PSO/PSA patients, abnormal activation of CD4 + Th17 cells and excessive production of cytokines like IL-17 cause skin and joint inflammation, while also inducing lung inflammation and fibrosis through the proliferation of fibroblasts and production of cytokines such as TNF-α, IL-6, IL-21, IL-22, and IL-23, leading to ILD [35, 36]. For ILD treatment accompanying PSO, reports indicate good responses to immunosuppressive drugs (Azathioprine and Secukinumab), emphasizing the importance of immunomodulation in treating ILD accompanying PSO [37, 38]. Our study identified a causal impact of PSA on ILD, with no such connection found between total PSO or PSV and ILD. In most cases, PSA develops a long time after the appearance of skin symptoms, and it is estimated that up to 30% of PSO may progress to PSA, suggesting that the progression of PSO may exacerbate immune abnormalities, potentially leading to ILD. This hypothesis, grounded in current research, necessitates further prospective studies to investigate the causal relationship between PSA and ILD and to elucidate underlying mechanisms. Additionally, the potential causal relationship between PSO/PSV and ILD warrants more exploration due to genetic heterogeneity, confounding factors, and potential errors introduced by weak instrument validity.

Our study’s strengths lie in the use of the MR method, which effectively minimizes the impact of confounding factors and reverse causation. Furthermore, we utilized IVs from multiple extensive GWAS datasets, enhancing the accuracy of our effect estimations. However, some limitations cannot be ignored. Firstly, our data is derived from European ancestry, lacking representation from other races, thus possibly lacking generalizability due to genetic heterogeneity. Secondly, ILD includes a variety of heterogeneous lung diseases, and due to data limitations, there is a lack of IVs corresponding to different ILD types, preventing exploration of potential causal impacts between PSO/PSA/PSV and different ILD types. Additionally, each ILD subtype has a unique pathogenesis that could potentially influence the accurate interpretation and applicability of our study results. Lastly, while we identified a causal impact of PSA on ILD, it is unclear whether this is specific to the phenotype or a result of more representative IVs for PSA. Consequently, therefore, multivariable MR analysis should be used for further exploration after obtaining larger genetic data on different ILD types.

## Conclusion

Our MR analysis did not identify a causal relationship between total PSO and ILD. However, there is a significant difference between the two phenotypes, PSA and PSV, manifested in a significant positive causal relationship between PSA and ILD, while no causal relationship was observed between PSV and ILD. In the reverse MR analysis, ILD does not increase the risk of PSO/PSA/PSV. Additionally, sensitivity analyses validated the robustness of the results. The implications of our findings are substantial. It not only strengthens the evidence of a causal link between PSA and ILD but also contributes significantly to our understanding of the genetic connection between these conditions. This insight is crucial for guiding clinical decisions, assessing risk, and informing public health strategies.

### Electronic supplementary material

Below is the link to the electronic supplementary material.

Supplementary Material 1

Supplementary Material 2

## Data Availability

All data generated or analyzed during this study are included in this published article and its supplementary information files.

## Abbreviations

PSO: Psoriasis
PSV: Psoriasis vulgaris
PSA: Psoriatic arthritis
GP: Guttate psoriasis
PP: Pustular Psoriasis
ILD: Interstitial lung disease
WHO: World Health Organization
MR: Mendelian randomization
GWAS: Genome-wide association studies
EBI: European Bioinformatics Institute
SNPs: Single nucleotide polymorphisms
IVW: Inverse variance weighted
CI: Confidence interval
DPLD: Parenchymal lung disease
CTD: Connective tissue diseases
