# Supplementary material for: A bidirectional two-sample Mendelian randomization study to evaluate the relationship between psoriasis and interstitial lung diseases
Source: BMC Pulm Med. 2024 Jul 9;24:330. doi: 10.1186/s12890-024-03146-y (PMC11234683; doi:10.1186/s12890-024-03146-y)
Supplement: Supplementary file 2 — Supplementary Material 2 [file 12890_2024_3146_MOESM2_ESM.docx]

**Supplementary Figures**


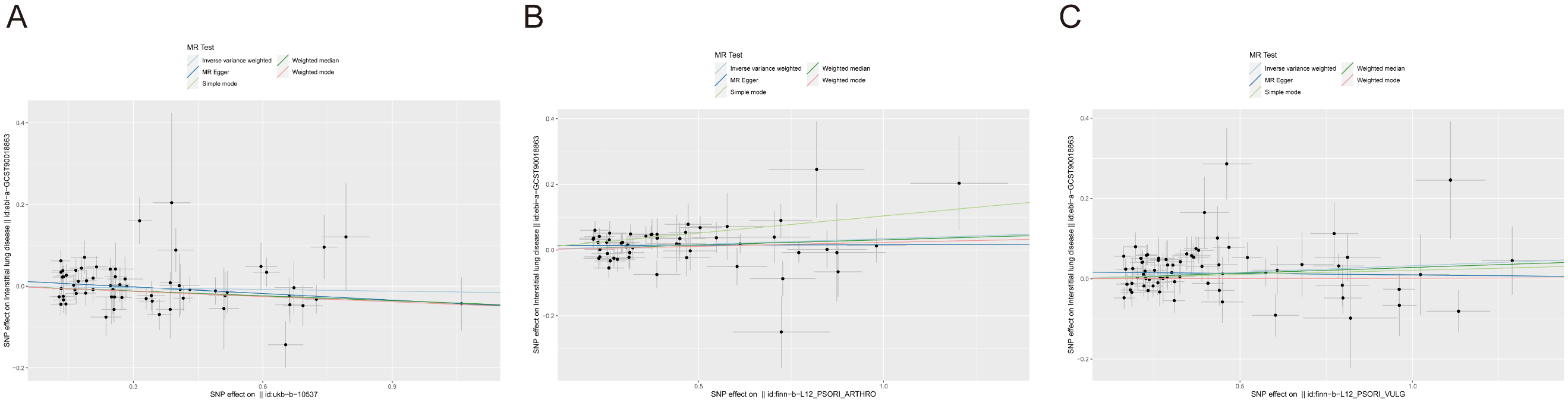


**Supplementary Figure 1.** Scatter plots of MR analysis. (A) PSO on ILD; (B) PSA on ILD; (C) PSV on ILD. ILD, interstitial lung disease; PSO, psoriasis; PSA, psoriatic arthritis; PSV, psoriasis vulgaris.


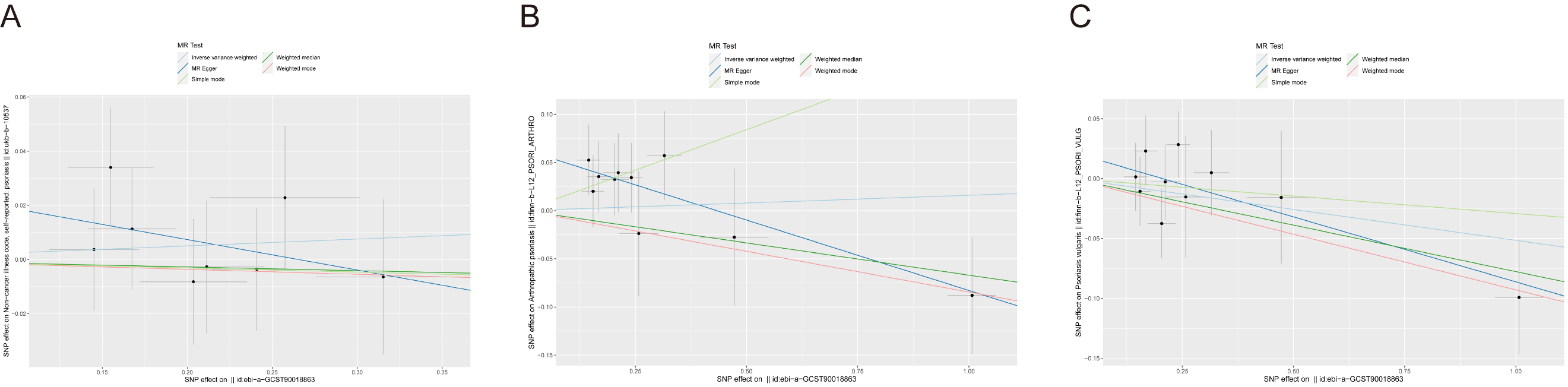


**Supplementary Figure 2.** Scatter plots of MR analysis. (A) PSO on ILD; (B) PSA on ILD; (C) PSV on ILD. ILD, interstitial lung disease; PSO, psoriasis; PSA, psoriatic arthritis; PSV, psoriasis vulgaris.


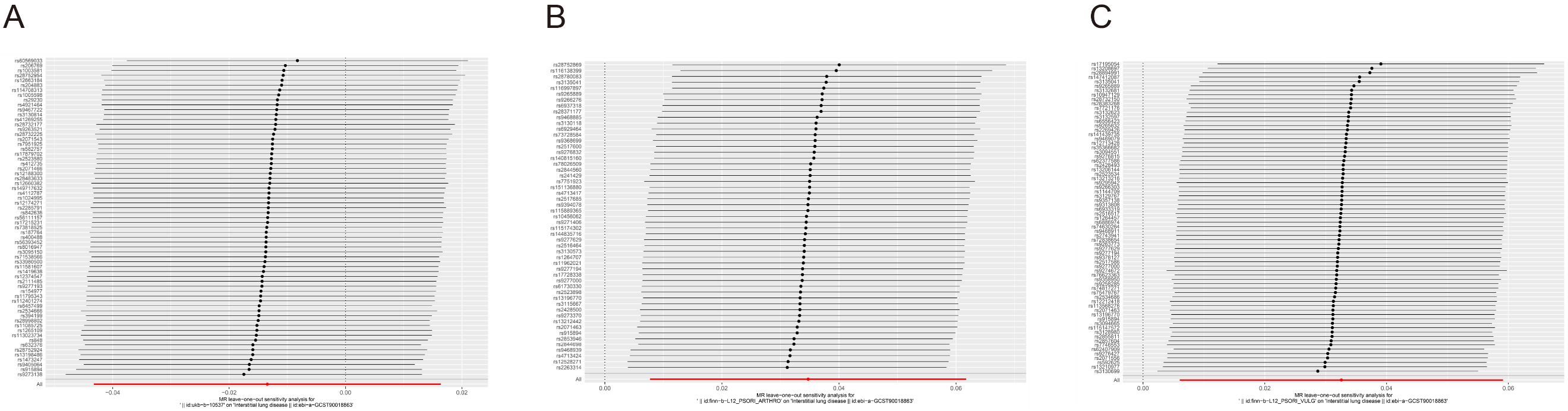


**Supplementary Figure 3.** Results of MR leave-one-out sensitivity analyses of total PSO, PSA and PSV on ILD. (A) PSO on ILD; (B) PSA on ILD; (C) PSV on ILD. ILD, interstitial lung disease; PSO, psoriasis; PSA, psoriatic arthritis; PSV, psoriasis vulgaris.


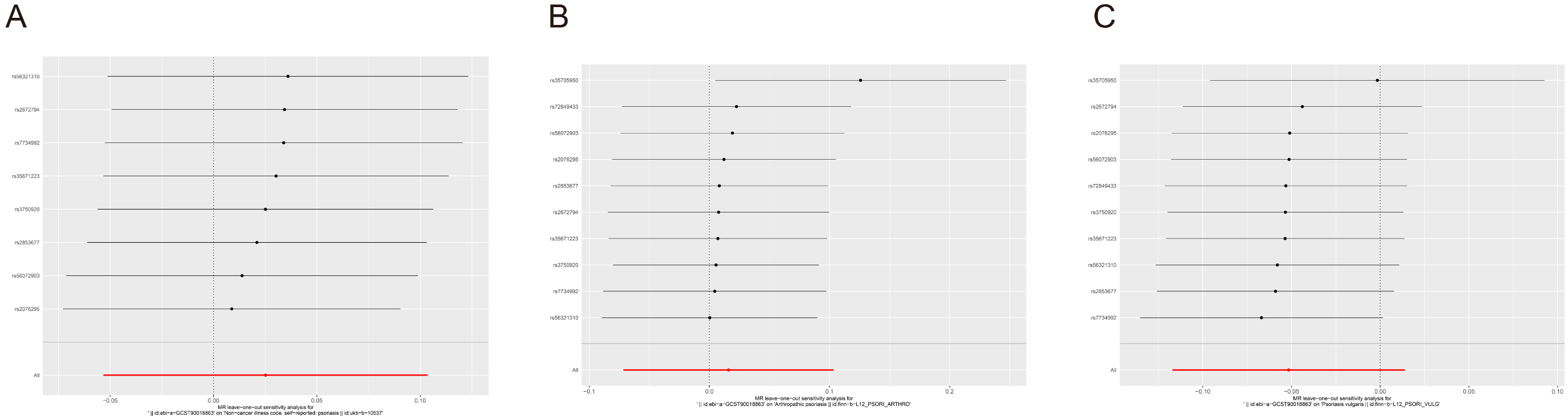


**Supplementary Figure 4.** Results of MR leave-one-out sensitivity analyses of ILD on total PSO, PSA and PSV on ILD. (A) PSO on ILD; (B) PSA on ILD; (C) PSV on ILD. ILD, interstitial lung disease; PSO, psoriasis; PSA, psoriatic arthritis; PSV, psoriasis vulgaris.
